# Criteria for referral of pediatric SARS-CoV-2 infection: a real-life experience in the pandemic era

**DOI:** 10.1186/s13052-020-00946-w

**Published:** 2020-12-07

**Authors:** Carlotta Montagnani, Elisabetta Venturini, Manuela L’Erario, Chiara Tersigni, Barbara Bortone, Leila Bianchi, Francesca Menegazzo, Giuseppe Indolfi, Elena Chiappini, Luisa Galli

**Affiliations:** 1 Infectious Diseases Unit, Meyer Children’s University Hospital, Florence, Italy; 2 Intensive Care Unit, Meyer Children’s University Hospital, Florence, Italy; 3 Department of Health Sciences, University of Florence, Florence, Italy; 4 Direzione Sanitaria, Meyer Children’s University Hospital, Florence, Italy; 5 Paediatric and Liver Unit, Meyer Children’s University Hospital, Florence, Italy

**Keywords:** SARS CoV-2, Children, Referral criteria, COVID-19

## Abstract

A practical guidance on the management of children with COVID-19 to insure homogeneous criteria for referral to a higher-level facility, according to the disease severity, is pivotal in the pandemic era. A panel of experts in pediatric infectious diseases and intensive care at the tertiary-care Meyer Children’s University Hospital, Florence, Italy, issued a practical document shared with Tuscany hospitals. The rationale was to target the referral for those children at risk of requiring an intensive support, since the above mentioned hospital has the pediatric intensive care unit. Overall, 378 patients between 0 and 19 years of age were diagnosed with COVID-19 infection in the Tuscany region with 24 (6.3%) hospitalizations. Only three children were centralized to Meyer Children’s University Hospital according to reported criteria. Considering that appropriate referral criteria have been associated with reduced mortality in other conditions, our document might be useful to improve outcomes of children with COVID-19.

To the Editor,

severe acute respiratory syndrome coronavirus 2 (SARS-CoV-2) is the causative agent of coronavirus disease 2019 (COVID-19) [1]. Due to the spreading of SARS-CoV-2 in Italy, a guidance on the management of children with COVID-19 is needed in order to insure homogeneous criteria for referral to a higher-level facility, according to the disease severity. A panel of experts in pediatric infectious diseases and intensive care, currently in a multidisciplinary group for COVID-19 care at the tertiary-care Meyer Children’s University Hospital, Florence, Italy, issued a practical document that has been shared with Tuscany hospitals. The rationale was to target the referral for those children at risk of requiring an intensive support, since the above mentioned hospital has the pediatric intensive care unit.

Overall, 378 patients between 0 and 19 years of age were diagnosed with COVID-19 infection in the Tuscany region, up to 31 July 2020. Of these, 24 (6.3%) have been hospitalized in Tuscany hospitals [2]. In particular, 14 children have been admitted to Meyer Children’s University Hospital and only 3 of them (21.4%) were centralized from other hospitals (two infants because under 3 months of age and one 14 years old age with a genetic disorder). None of the children were admitted in intensive care unit.

In fact, according to the currently available data, COVID-19 in children usually presents as a asymptomatic/pauci-symptomatic disease. Asymptomatic cases do not require further evaluation unless clinical deterioration occurs. If present, clinical manifestations include fever (44–50%), dry cough (38%), asthenia. Other signs/symptoms are nasal congestion, rhinitis, headache, diarrhea, feeding difficulties [1, 3]. However, mild and severe cases are also described in children and disease severity can be classified as follows.

- Asymptomatic
- Pauci-symptomatic/uncomplicated case: fever and/or asthenia with mild upper respiratory signs, such as coryza, nasal obstruction
- Moderate case: fever and/or asthenia and/or respiratory signs/symptoms, such as cough, mild distress with polypnea and/or difficulty in feeding, signs of dehydration
- Severe case: fever and/or cough, plus at least one of the following:
- SpO2 < 92% on finger pulse oximeter taken at rest
  - Labored breathing (moaning, nasal flattering, sternal, clavicular and internal recesses.
  - ribs), cyanosis, intermittent apnea.
  - Tachypnea, in apyrexia and absence of crying (respiratory rate > 60 breaths/minute < 3 months; > 50 breaths /minute 3–12 months; > 40 breaths /minute 1–5 years; > 30 breaths/ minute > 5 years).
  - Systemic signs of worsening: lethargy, inability to feed/drink, convulsions.
  - Suspected sepsis.
  - Shock or other organ failure requiring care.

It should be underlined that the early identification of risk factors and warning indicators for severe and critical disease is of paramount importance. These includes the following criteria:

- Age < 3 months.
- Underlying diseases (e.g. congenital heart disease, bronchopulmonary dysplasia, respiratory tract malformation, cystic fibrosis, hemoglobinopathies, severe malnutrition, abnormal hemoglobin, congenital or acquired immunodeficiencies, etc.)
- Respiratory rate increasing despite intravenous hydration and oxygen therapy with nasal cannula/mask after 2 h of treatment.
- Poor mental reaction and drowsiness.
- Lactate increasing progressively.
- Bilateral or multiple lobe lung infiltrates, pleural effusion, rapid progression of radiological changes.
- Acute respiratory distress syndrome (ARDS) [4]

According to the present document, referral of patients with SARS-Cov-2 infection is not necessary in asymptomatic or uncomplicated cases. In moderate cases, referral should be established on the basis of criteria reported in Table 1. The bedside PEWS score is a useful tool to detect changing in the clinical picture [5]. It is appropriate in the presence of warning indicators or if the local hospital is unable to guarantee an isolation room or the level of care required. All severe cases should be early referred to a tertiary-care hospital with a pediatric intensive-care facility. Considering that appropriate referral criteria have been associated with reduced mortality in other conditions [6], our document might be useful to improve outcomes of children with COVID-19.

**Table 1:** Referral criteria for children with COVID-19

	Patient assessment	Supportive care	Setting of care	Referral
**Asymptomatic infection**	None	None	Discharge at home, refer to the family pediatrician with indications on isolation	No
**Pauci-symptomatic/ uncomplicated case**	Oxygen saturation	None In case of fever > 38 °C: paracetamol	Discharge at home, refer to the family pediatrician with indications on isolation	No
**Moderate case**	• Monitor vital signs (Bedside-PEWS) • Blood tests: full blood count, C-reactive protein, erythrocyte sedimentation rate, procalcitonin, liver enzymes, lactate dehydrogenase, creatine phosphokinase, creatinine, electrolytes, hemogasanalysis, coagulation tests (prothrombin time, partial thromboplastin time, fibrinogen, D-dimers, INR) • Pulmonary ultrasound (if available) • Chest x-ray in selected cases • Other tests based on the clinical picture	• Airway suction in case of obstruction • Oxygen therapy using nasal cannulas or facial mask with Venturi system (if oxygen saturation in air < 95%) • Intravenous access, adequate fluid and caloric intake based on hydration status • Give paracetamol in case of fever > 38 °C	Hospitalization, isolation in single room with closed door	If the score (Bedside-PEWS) is not improved or increases after 2 h since oxygen and hydration support, refer the patient to a tertiary care hospital Refer to a tertiary-care hospital if: - presence of alarm criteria - needing for Venturi mask or High Flow Nasal Cannula to maintain SpO2 > 95% - relevant hematological alterations Referral should always be agreed with the infectious disease specialist
**Severe case**	• Monitor vital signs (Bedside-PEWS) in order to early identify warning indicators: - respiratory rate > 60 breaths/minute < 3 months; > 50 breaths /minute 3–12 months; > 40 breaths /minute 1–5 years; > 30 breaths/ minute > 5 years - SpO2 92–93% with FiO_2_ ≥ 40% - Poor mental reaction and drowsiness - Increases of liver tests, muscular and cardiac enzymes - Metabolic acidosis - Bilateral interstitial infiltrates, pleural effusion on chest x-ray; rapid progression of radiological findings • Blood tests: full blood count, C-reactive protein, erythrocyte sedimentation rate, procalcitonin, ferritin, liver enzymes, lactate dehydrogenase, creatine phosphokinase, creatinine, electrolytes, hemogasanalysis, coagulation tests (prothrombin time, partial thromboplastin time, fibrinogen, D-dimers, INR), myocardial enzymes • Pulmonary ultrasound (if available) • Chest x-ray • Computer tomography scan in selected cases • Other tests based on the clinical picture	• Airway suction in case of obstruction • Oxygen therapy using nasal cannulas or facial mask with Venturi system or High Flow Nasal Cannula (target oxygen saturation > 95%) • Intravenous access, adequate fluid and caloric intake based on hydration status. Monitor urinary output. • Give paracetamol in case of fever > 38 °C • Avoid empiric antibiotic treatment if no evidence of bacterial infection (consult an infectious disease specialist or refer to hospital guidelines)	Hospitalization, isolation in negative pressure room or, if not available, in single room with closed door Intensive care admission indicated if warning signs does not improve after 2 h of patient support	Refer directly the patient to the tertiary care hospital Referral should always be agreed with the infectious disease and intensive care specialists

## Data Availability

Data sharing is not applicable to this article as no datasets were generated or analysed during the current study.

## Abbreviations

ARDS: Acute respiratory distress syndrome
COVID-19: Coronavirus disease 2019
SARS-CoV-2: Severe acute respiratory syndrome coronavirus 2

## References

[1] World Health Organization (2020). Clinical management of severe acute respiratory infection (SARI) when COVID-19 disease is suspected. Interim guidance.

[2] Azienda Regionale di Sanità. Rapporto sui casi di infezione da SARS-CoV-2 in Toscana. https://www.ars.toscana.it/images/qualita_cure/coronavirus/rapporti_Covid-19/Rapporto_COVID-19_31_LUGLIO_2020_DEF.pdf Last accessed: 15 August 2020.

[3] Centers for Disease Control and Prevention. Interim Infection Prevention and Control Recommendations for Patients with Suspected or Confirmed Coronavirus Disease 2019 (COVID-19) in Healthcare Settings. Available at: https://www.cdc.gov/coronavirus/2019-ncov/infection-control/control-recommendations.html. Last accessed: 15 August 2020.

[4] Khemani RG, Smith LS, Zimmerman JJ, Erickson S (2015). Pediatric Acute Lung Injury Consensus Conference Group. Pediatric acute respiratory distress syndrome: definition, incidence, and epidemiology: proceedings from the Pediatric Acute Lung Injury Consensus Conference. Pediatr Crit Care Med.

[5] Parshuram CS, Duncan HP, Joffe AR, Farrell CA, Lacroix JR, Middaugh KL (2011). Multicentre validation of the bedside paediatric early warning system score: a severity of illness score to detect evolving critical illness in hospitalised children. Crit Care.

[6] Ostermann M, Vincent JL (2019). How much centralization of critical care services in the era of telemedicine?. Crit Care.

